# Efficacy and safety of galcanezumab for preventive treatment of migraine: a systematic review and meta-analysis

**DOI:** 10.1007/s00415-020-09707-5

**Published:** 2020-01-31

**Authors:** Xiuyuan Zhao, Xiaolin Xu, Qingyun Li

**Affiliations:** 1 Tianjin Medical University, Tianjin, China; 2 Neurology, Tianjin Huanhu Hospital, Tianjin, China

**Keywords:** Galcanezumab, Migraine, Prophylaxis, Meta-analysis

## Abstract

**Objective:**

This meta-analysis aimed to systematically evaluate the effectiveness and safety of galcanezumab in the prophylactic treatment of adult migraine.

**Methods:**

A systematic literature search was performed to identity randomized-controlled trials (RCTs). The primary outcome was the decline in the number of monthly migraine days (MMDs). Secondary outcomes included the reduction of monthly acute migraine‑specific medication days (MSMDs), the number of participants showing a reduction in MMDs from baseline of ≥ 50%, ≥ 75%, and 100%, the incidence of adverse events (AEs), and the number of participants developing anti-drug antibodies (ADAs) to galcanezumab. We calculated the mean difference (MD), relative risk (RR), and 95% confidence intervals (CIs) for these outcomes.

**Results:**

Among the five included trials, galcanezumab given at doses of 120, 150, 240, and 300 mg was superior to placebo for both MMDs and secondary outcomes. The degree of AEs in all group was mild. Notably, no significant differences were found in the occurrence of AEs and ADAs between the galcanezumab and placebo groups.

**Conclusion:**

Galcanezumab is a safe and effective treatment for adult patients with episodic and chronic migraine.

## Introduction

Migraine is a common primary headache disorder that has been regarded as one of the most disabling disorders affecting millions of people worldwide. Headaches manifest as pain of varying intensity characterized by repeated, moderate, or severe, unilateral, or bilateral pulsating headaches lasting for hours and possibly days often combined with autonomic dysfunctions such as nausea, vomiting, photophobia, and phobia. Some patients also experience prodromal and/or postdromal phase-like symptoms. Headaches persisting for 15 days or more (over 3 months for 8 consecutive days in a month) are considered as chronic migraines [1]. According to statistics, in 2016, nearly 1.04 billion individuals suffered from migraine. By 2016, migraine accounted for 45.1 million years of life lived with disability (YLDs), representing an increase of 51.2% from 29.8 million YLDs in 1990 [2]. Chronic migraine is the major cause of more severe headache-related disability, when compared to episodic migraine, although it affects merely 1–3% of the worldwide population [3–5]. Patients with migraines have higher expenditures on outpatient, emergency, and prescription drug than those without this disorder [6]. Individuals with chronic migraine have to bear greater headache-related direct, indirect, and total costs [7].

The etiology and pathogenesis of migraine remains incompletely understood. However, some neurotransmitters and vasoactive substances, such as nitric oxide (NO), serotonin (5-hydroxytryptamine; 5-HT), and calcitonin gene-related peptide (CGRP), have been identified to be implicated in the initiation of migraine. The 5-HT receptor agonists, e.g., ergotamine and triptans, and CGRP receptor antagonists have been proven to be effective for the treatment of migraine attacks [1, 8]. However, these drugs can cause medication overuse headache, hepatotoxicity, and cardiovascular and central side effects [9]. A survey revealed that more than 70% of patients using these drugs stopped or switched treatment due to drug side effects and intolerance. While so far, monoclonal antibodies against CGRP were almost devoid of serious adverse reactions [9, 10].

Thus, anti-CGRP monoclonal antibodies are used as the prophylactic treatments for migraine. Galcanezumab was authorized in the USA in May 2018. In this meta-analysis, we collected and analyzed RCTs of galcanezumab in the prevention of migraine in adults. The results of this analysis are expected to provide evidence-based date for prophylactic managements of migraine.

## Methods

### Search strategy

This meta-analysis was conducted according to the Preferred Reporting Items for Systematic Reviews and Meta-Analyses (PRISMA). We searched for relevant RCTs on the PubMed, the Cochrane Library, Web of Science, and Clinicaltrail.gov using the following keywords: migraine and galcanezumab or LY2951742. The last search date was March 3, 2019. During the search, two authors independently read the whole articles and inspected the reference lists. Any divergence was resolved by discussion between the authors.

### Inclusion and exclusion criteria

We included articles that met the following criteria: (1) the study was a randomized-controlled trial (RCT) on calcitonin gene-related peptide monoclonal antibody (CGRP-mAb) for migraine prophylaxis; (2) the patients were diagnosed with migraine according to the International Classification of Headache Disorders, third edition (ICHD-3, beta version) or the International Classification of Headache Disorders (ICHD-II); (3) no limitations on the time of publication, and blind or publication types. Studies were excluded when one of the following situations occurred: (1) the subjects were non-adults with migraine; (2) the CGRP-mAbs were administered as adjuvant drugs; and (3) studies were not RCTs.

### Data extraction

Two investigators independently assessed the selected studies and extracted the following information: main author, completion date, study design (methods of randomization, allocation and blinding, type of migraine, dose of intervention, route of administration, frequency of injection and course of treatment), basic information of the research objects (number of participants, age range of participants, number of the males and females, and baseline characteristics), inclusion and exclusion criteria, primary and secondary outcome measures, and adverse events. This metanalysis conformed to the principles of the Cochrane Handbook for Systematic Reviews of Interventions. Any discrepancies in the results were resolved through consensus between investigators.

### Data analysis

Since the extent of heterogeneity may affect the results and conclusions of a meta-analysis, the Chi-square test was used to assess the statistical heterogeneity. It was suitable to use the fixed-effect model to analyze whether *I*^2^ < 50%, which meant that there was no significant heterogeneity. Otherwise, heterogeneity was regarded as unacceptable, and a random-effects model or subgroup analysis was considered. Publication bias was determined using funnel plots. Continuous outcomes were analyzed using mean differences (MD) and 95% confidence intervals (CIs), while dichotomous outcomes were analyzed using relative risk (RR) and 95% CIs. * P* < 0.05 was regarded as statistically significant. All data analyses were performed using Review Manager 5.3.

## Results

### Selection and characteristics of studies

**Fig. 1:**
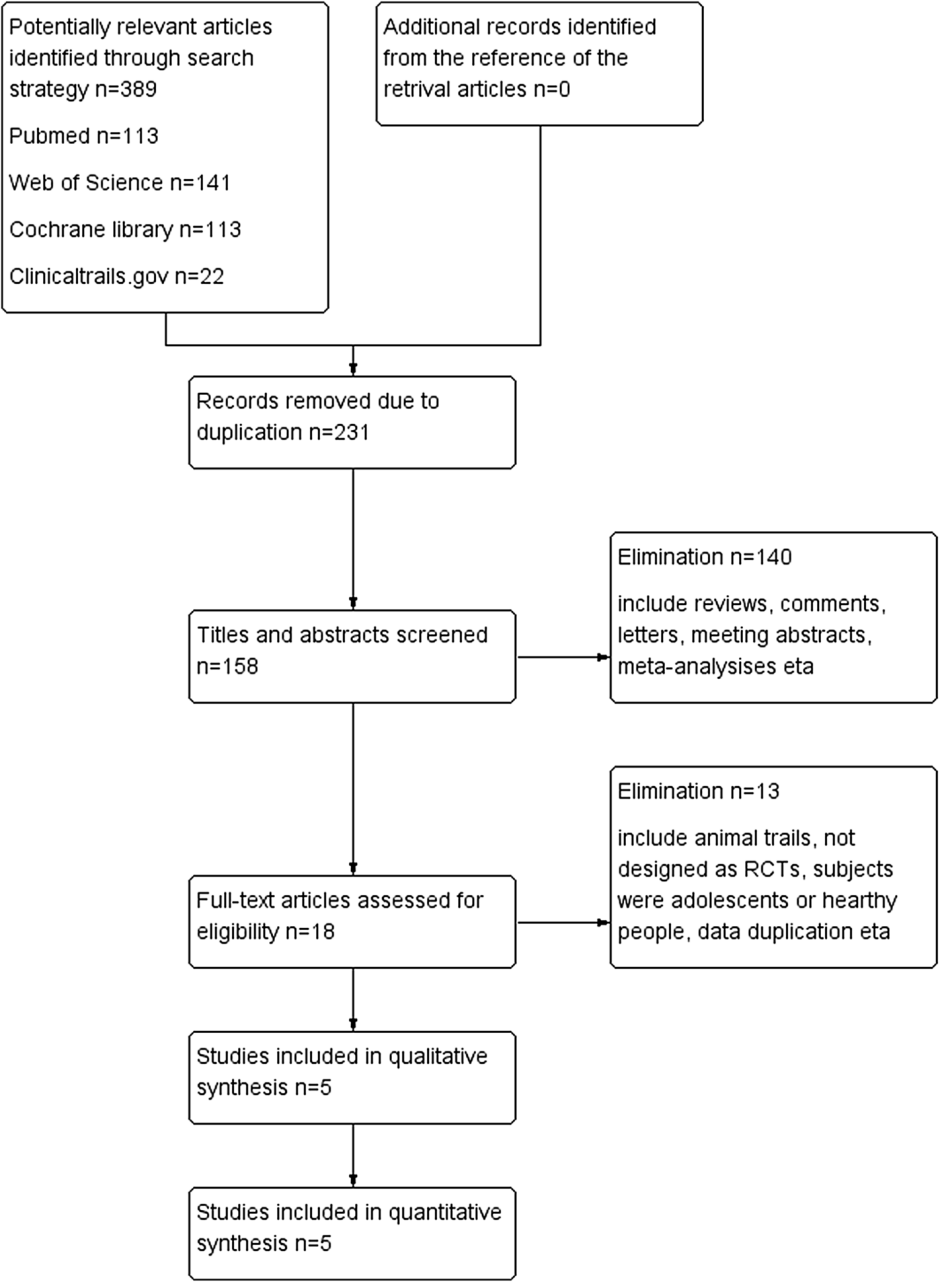
Flow diagram of the literature selection process

A total of 389 compositions were identified in the preliminary search. Among them, five studies were adopted in the analysis. The other studies were excluded for various reasons. Details of the screening procedure are presented in Fig. 1. All included studies were two-phase II and three-phase III trials, and were multicenter, randomized, double-blinded and placebo-controlled trials. The included trails covered a total of 3565 patients with episodic or chronic migraine. Different doses of galcanezumab were reported: 5, 50, 120, 150, 240, and 300 mg. The baseline characteristics of these studies and participants are summarized in Tables 1 and 2.

**Table 1:** Main characteristics of the enrolled studies

Study	Study design	Diagnosis	Enrollment	Interventions	Course of treatment	Primary outcome measures
Dodick et al. [12]	Phase II	Episodic migraine	190 subjects	Galcanezumab 150 mg Placebo SC once every 14 days	12 weeks	Mean change from baseline in the number of migraine headache days (MHD)
Skljarevski et al. [13]	Phase IIb	Episodic migraine	414 subjects	Galcanezumab 5 mg/50 mg/120 mg/300 mg Placebo SQ once every 28 days	12 weeks	Mean change from baseline in the number of MHD
Detke et al. [16]	Phase III (REGAIN)	Chronic migraine	1,113 subjects	Galcanezumab 120 mg/240 mg Placebo SC once every 28 days	12 weeks	Overall mean change from baseline in the number of monthly MHD
Stauffer et al. [14]	Phase III (EVOLVE-1)	Episodic migraine	862 subjects	Galcanezumab 120 mg/240 mg Placebo SC once every 28 days	24 weeks	Overall mean change from baseline in the number of monthly MHD
Skljarevski et al. [13]	Phase III (EVOLVE-2)	Episodic migraine	986 subjects	Galcanezumab 120 mg/240 mg Placebo SC once every 28 days	24 weeks	Overall mean change from baseline in the number of monthly MHD

**Table 2:** Main characteristics of the participants

	Dodick et al. [12		Vladimir Skljarevsk (2018)		Detke et al. [16]			Stauffer et al. [14]			Skljarevski et al. [13]		
	Galcanezumab 150 mg	Placebo	Galcanezumab	Placebo	Galcanezumab 120 mg	Galcanezumab 240 mg	Placebo	Galcanezumab 120 mg	Galcanezumab 240 mg	Placebo	Galcanezumab 120 mg	Galcanezumab 240 mg	Placebo
	*n* = 107	*n* = 110	*n* = 273	*n* = 137	*n* = 278	*n* = 277	*n* = 558	*n* = 213	*n* = 212	*n* = 433	*n* = 231	*n* = 223	*n* = 461
Age (years)	40·9 (11·4)	41·9 (11·7)	40.6 (11.9)	39.5 (12.1)	39.7 (11.9)	41.1 (12.4)	41.6 (12.1)	40.9 (11.9)	39.1 (11.5)	41.3 (11.4)	40.9 (11.2)	41.9 (10.8)	42.3 (11.3)
Women in participants	88 (82%)	96 (87%)	231 (84.6%)	109 (79.6%)	237 (85%)	226 (82%)	483 (87%)	181 (85%)	175 (82.6%)	362 (83.6%)	197(85.3%)	191(85.7%)	393(85.3%)
White in participants	76 (71%)	74 (67%)			223 (80%)	224 (81%)	432 (77%)	169 (79.3%)	165 (77.8%)	356 (82.2%)	166(71.9%)	152(68.2%)	325(70.5%)
Body-mass index (kg/m^2^)	29·44 (6·3)	29·03 (7·5)			26.4 (5.5)	26.7 (5.2)	26.9 (5.6)	27.8 (5.3)	28.6 (5.7)	28.6 (5.5)			
Migraine illness duration (years)					20.4 (12.7)	20.1 (12.7)	21.9 (12.9)	21.1 (13.0)	19.3 (11.9)	19.9 (12.3)	19.93 (11.7)	20.01 (12.1)	21.2 (12.8)
MHD (mo)	6·7 (2·4)	7·0 (2·5)	6.7 (2.6)	6.6 (2.7)	19.4 (4.3)	19.2 (4.6)	19.6 (4.6)	9.2 (3.1)	9.1 (2.9)	9.1 (3.0)	9.07 (2.9)	9.06 (2.9)	9.2 (3.0)
MHD with acute medication use (mo)					15.1 (6.3)	14.5 (6.3)	15.5 (6.6)	7.4 (3.7)	7.3 (3.3)	7.4 (3.5)	7.47 (3.3)	7.47 (3.3)	7.6 (3.4)
MHD category ≥ 8								140 (65.7%)	139 (65.6%)	285 (65.8%)	154 (66.7%)	151 (67.7%)	307 (66.6%)
Migraine attacks (mo)	4·9 (1·6)	5·0 (1·7)	4.7 (1.6)	4.7 (1.5)				5.6 (1.7)	5.7 (1.8)	5.8 (1.7)	5.54 (1.8)	5.66 (1.8)	5. 7 (1.8)
History of aura	46 (43%)	44 (40%)			153 (55%)	141 (51%)	310 (56%)						
Headache (d/mo)					21.2 (4.0)	21.4 (4.1)	21.5 (4.1)				10.56 (3.4)	10.74 (3.7)	10.7 (3.5)
Headache (h/mo)					144.7 (85.4)	145.9 (93.4)	145.1 (95.1)	59.9 (40.0)	65.0 (60.2)	58.8 (39.4)			
Prior preventive treatment					211 (76%)	220 (79%)	435 (78%)a	133 (62.4)	125 (59.0)	257 (59.4)	157 (68.0%)	144 (64.6%)	298 (64.6%)
Acute headache medication overuse					178 (64%)	177 (64%)	353 (63%)	67 (31.5%)	62 (29.3%)	146 (33.7%)			
MSQ RF-R score					39.3 (17.3)	38.9 (17.3)	38.4 (17.2)	51.4 (16.2)	48.8 (16.8)	52.9 (15.4)	52.5 (14.8)	51.7 (16.3)	51.4 (15.7)
PGI-S score					4.8 (1.2)	4.9 (1.3)	4.9 (1.2)	4.4 (1.1)	4.5 (1.1)	4.2 (1.1)	4.1 (1.2)	4.2 (1.2)	4.3 (1.2)

### Risk of bias

**Fig. 2:**
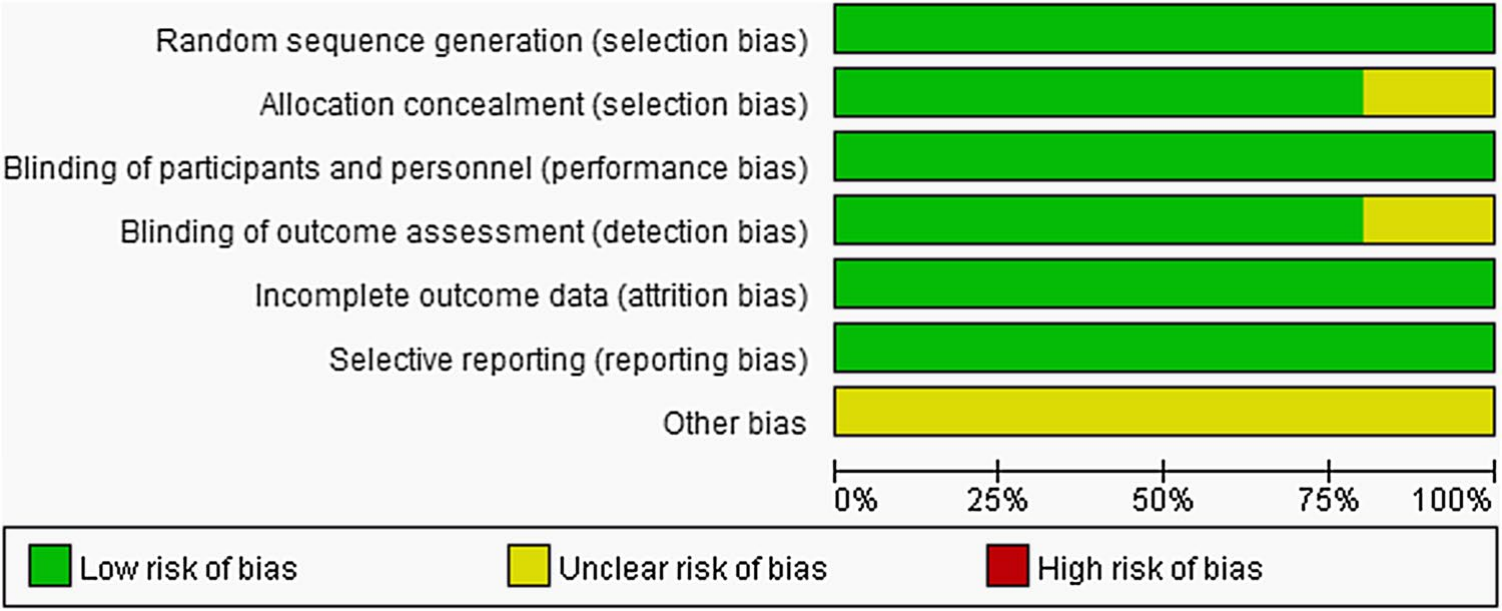
Risk of bias for the included trials

**Fig. 3:**
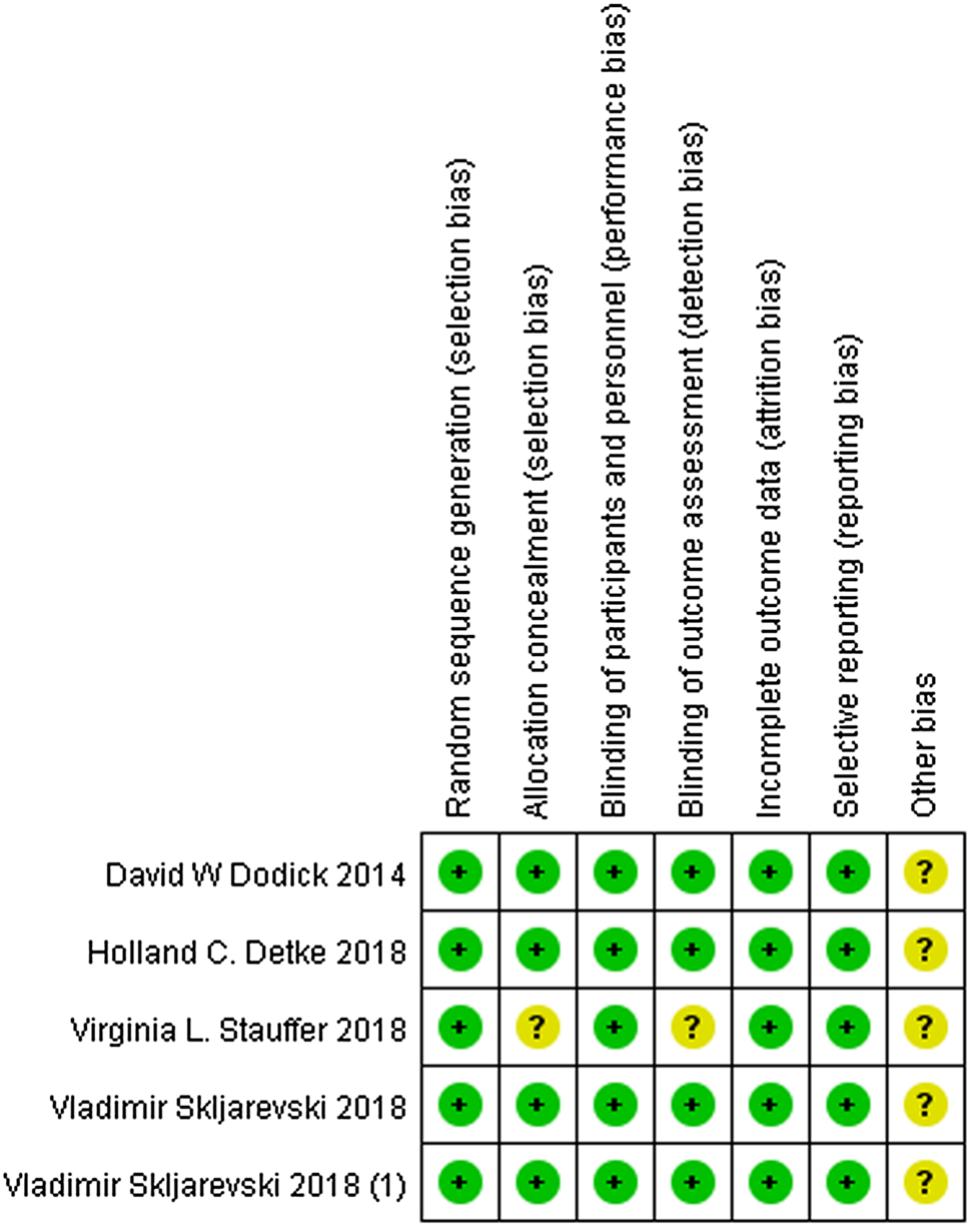
Risk of bias summary for the included trials

Bias were assessed according to the Cochrane Handbook of Systematic Review. The details are presented in Figs. 2 and 3. All participants in the five studies were randomly assigned to groups via a computer-generated random sequence with an interactive web-response system. One trial reported that the pharmacists were unmasked, but they did not participate in any other aspect of the study other than the preparation and inventory of drugs. Another trial did not expound the allocation concealment and blinding of the outcome assessment. All five trials recorded the loss of follow-up of patients. Furthermore, each pre-defined outcome was explained. All trials that met the inclusion criteria were enrolled in this meta-analysis.

## Efficacy evaluation

### Monthly migraine days (MMDs) and monthly acute migraine-specific medication days (MSMDs)

**Fig. 4:**
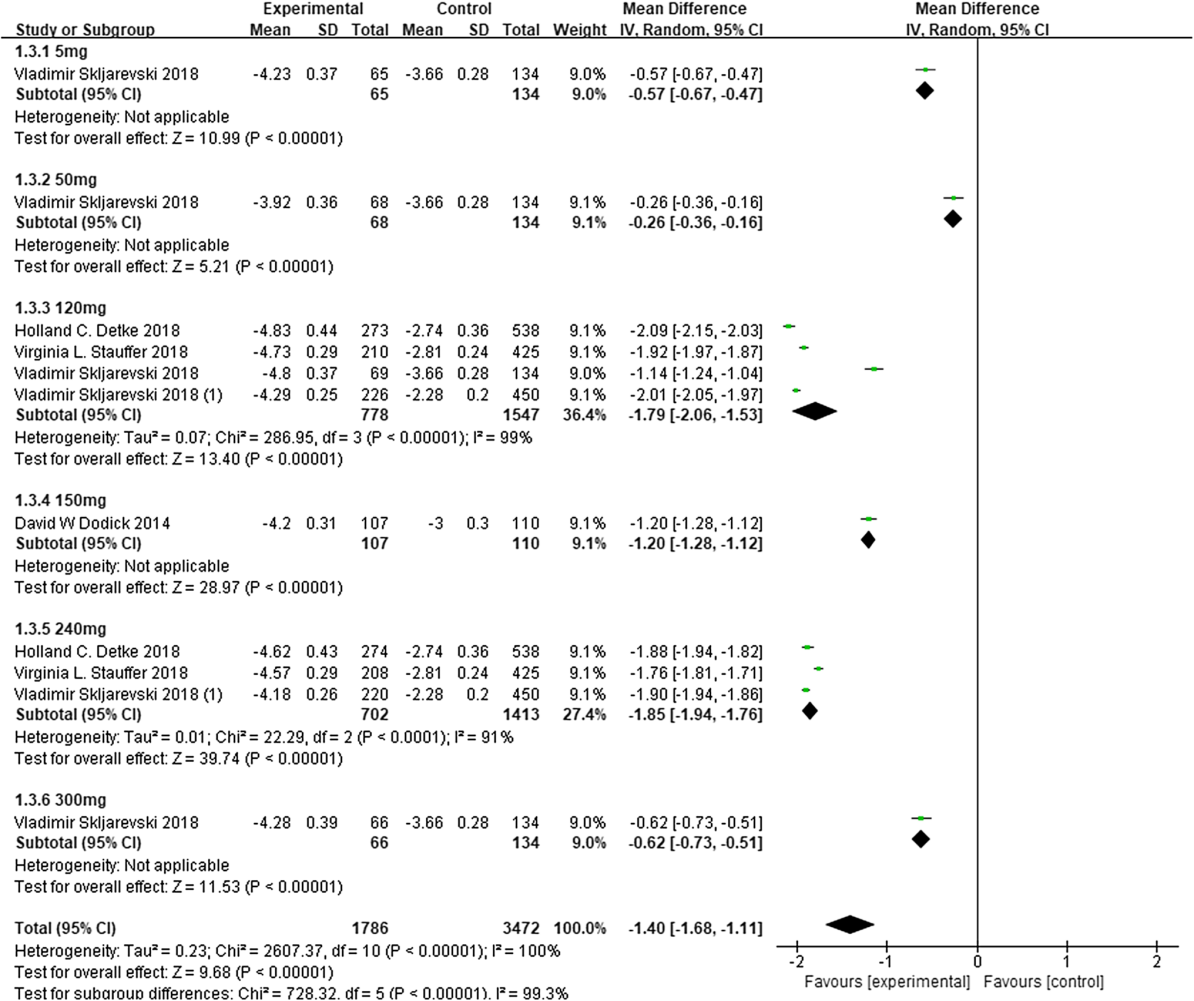
Change from baseline in MMDs. *MD* mean difference, *CI* confidence interval

**Fig. 5:**
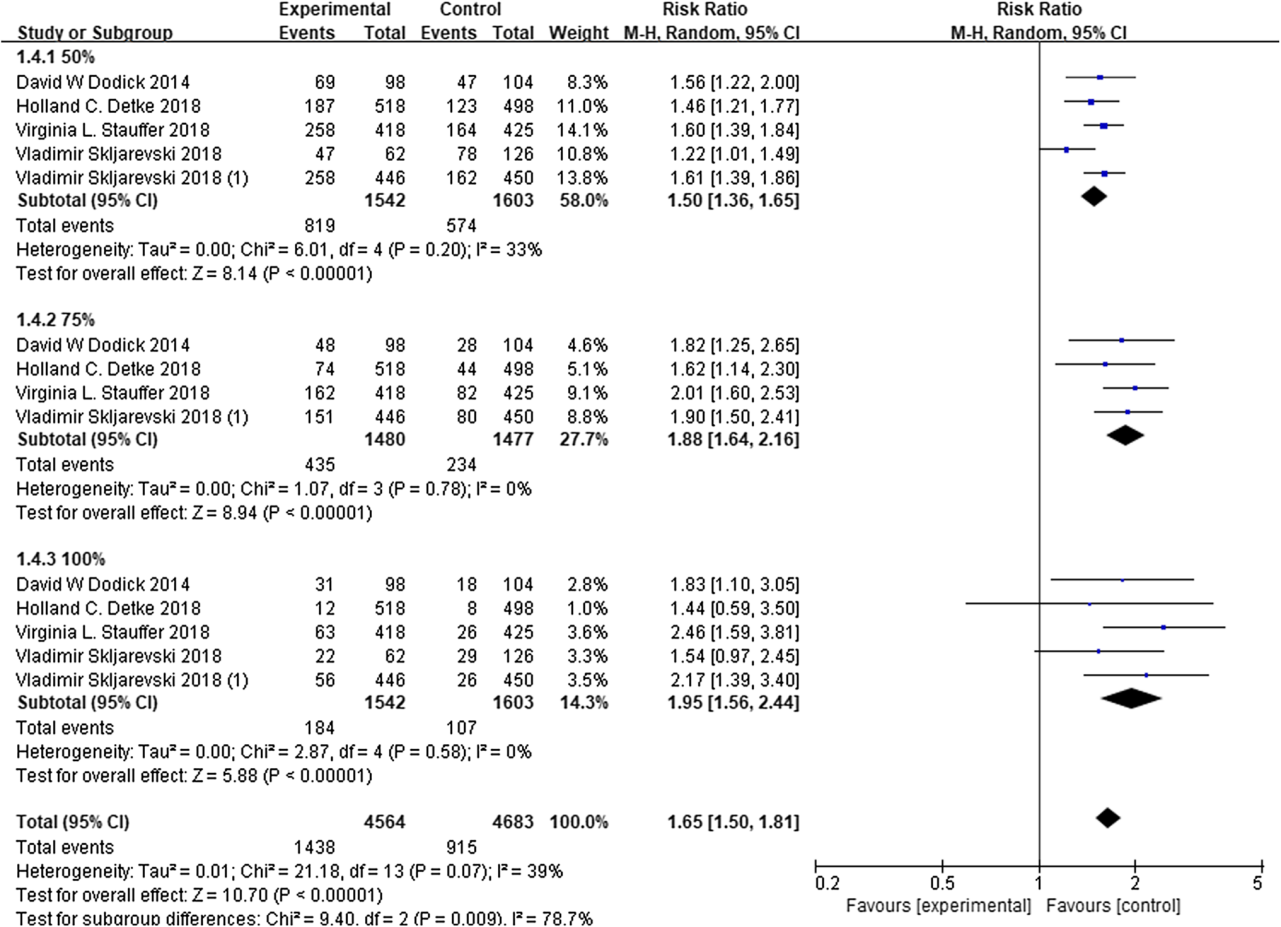
**a **Funnel plot 1. Funnel plot of the reduction in MMDs, **b **Funnel plot 2. Funnel plot for the 50%, 75% and 100% responder rates of the reduction from baseline in MMDs, **c **Funnel plot 3. Funnel plot of adverse events

Subgroup analysis performed with regard to the dose demonstrated that galcanezumab at 120, 150, 240, and 300 mg significantly reduced MMDs (120 mg: MD − 1.79, 95% CI − 2.06 to − 1.53, *P* < 0.00001; 150 mg: MD − 1.20, 95% CI − 1.28 to − 1.12, *P* < 0.00001; 240 mg: MD − 1.85, 95% CI − 1.94 to − 1.76, *P* < 0.0001; 300 mg: MD − 0.62, 95% CI − 0.73 to − 0.51, *P* < 0.00001; Fig. 4). There was notable heterogeneity in the overall results (*P* < 0.00001, *I*^2^ = 100%), while by removing any single hazard ratio from the meta-analysis, the sensitivity analysis did not substantively alter the overall result. The inverse funnel plot, which evaluated the risk of publication bias, was approximately symmetrical indicating no significant publication bias in the results (Fig. 5a). Studies in our meta-analysis also revealed that the reduction in MSMDs for galcanezumab of 120 and 240 mg vs. placebo was at a statistically significant level.

## The 50%, 75%, and 100% responder rate

**Fig. 6:**
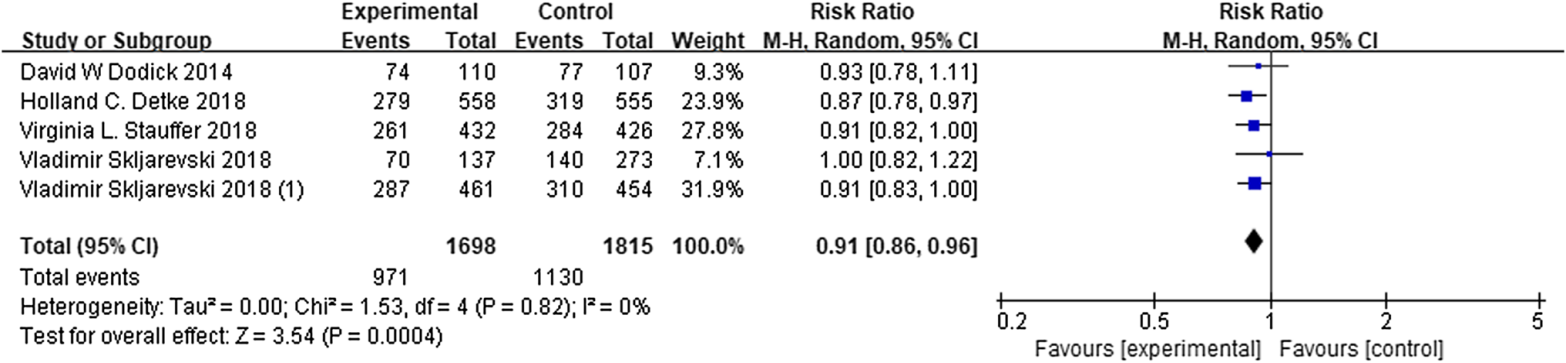
The ≥ 50% reduction change from baseline in MMDs. *RR* risk ratio, *CI* confidence interval

Compared to the placebo group, patients in the galcanezumab group were more likely to represent a significant increase of 50%, 75%, and 100% in responder rates of the reduction from baseline in MMDs (50%: RR 1.50, 95% CI 1.36–1.65, *P* = 0.20; 75%: RR 1.88, 95% CI 1.64–2.16, *P* = 0.78; 100%: RR 1.95, 95% CI 1.56–2.44, *P* = 0.58; Fig. 6). However, the meta-analysis revealed a non-significant heterogeneity among the included trials (*P* = 0.07, *I*^2^ = 39%). The inverse funnel plot (Fig. 5b) presented a low level of publication bias.

## Functional measurement

The phase II study of galcanezumab assessed the migraine-specific quality of life using the Migraine-Specific Quality of Life (MSQL) questionnaire and the Headache Impact Test™ (HIT-6). However, those data were not underwent formal statistical analyses. At the phase IIb study, Vladimir Skljarevski et al. conducted a post hoc secondary analyses with the same questionnaires. The results demonstrated that the change in MHD was concerned with the improvements in MSQL and the decline in HIT-6 scores. In the phase III studies, the investigators mainly focused on the change in Migraine-Specific Quality of Life questionnaire role function restrictive domain (MSQ RFR). It was found that both doses of galcanezumab led to a greater improvement in scores, when compared to placebo, i.e., the treatment with galcanezumab was associated with the reduction in functional impairment [11].

## Adverse events

**Fig. 7:**
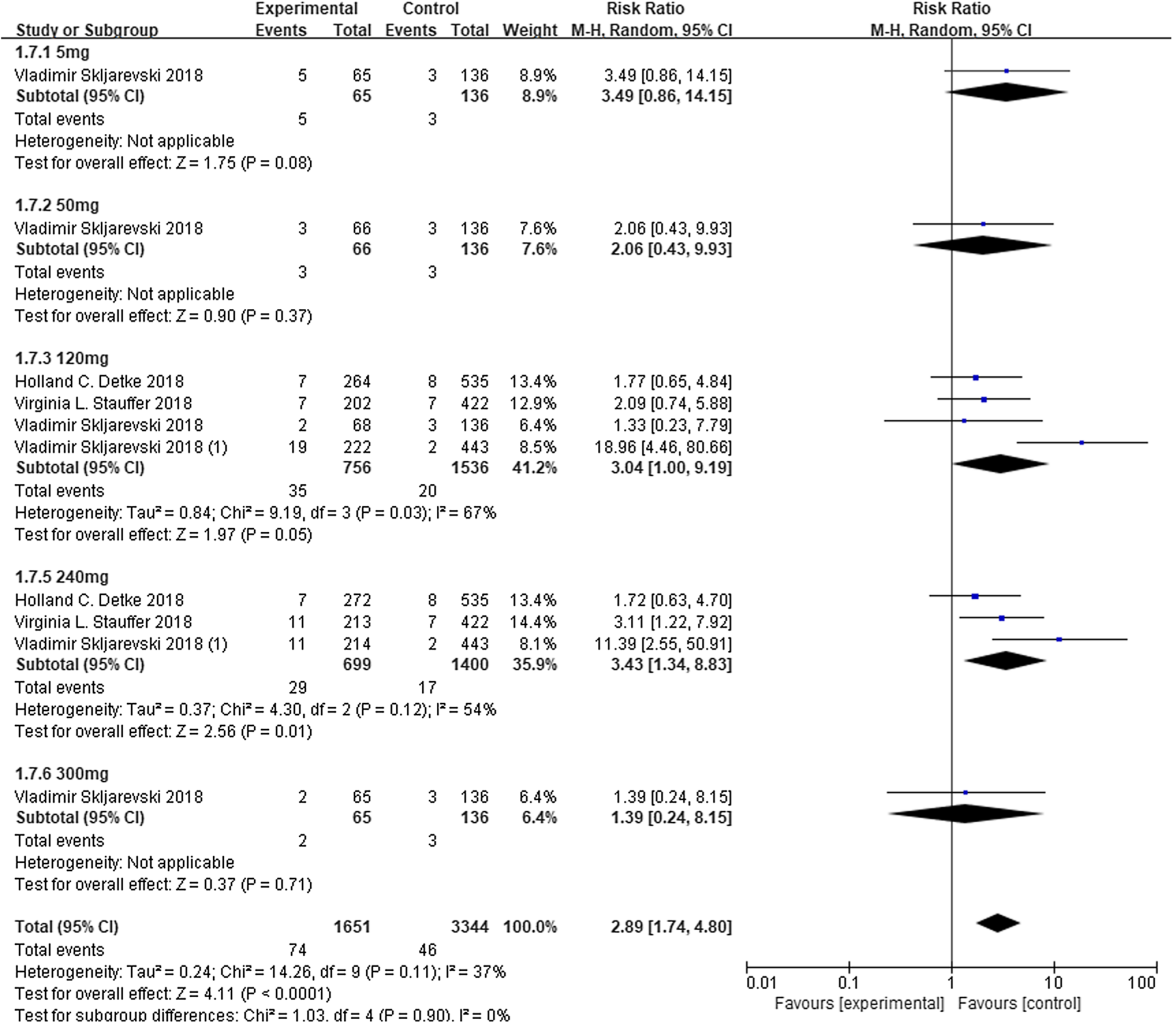
Adverse events for galcanezumab. *RR* risk ratio, *CI* confidence interval

A total of 2998 patients in all trials reported adverse events to different degrees. The total adverse events observed in patients with galcanezumab were not significantly different from those that occurred in the placebo groups, based on the meta-analysis (RR 0.91, 95% CI 0.86–0.96, *P* = 0.82; Fig. 7). Furthermore, there were no significant heterogeneity (*P* = 0.82, *I*^2^ = 0%) and no obvious publication bias in the results, which are presented in Fig. 5c. These findings suggest that galcanezumab is safe for migraine prophylaxis.

**Fig. 8:**
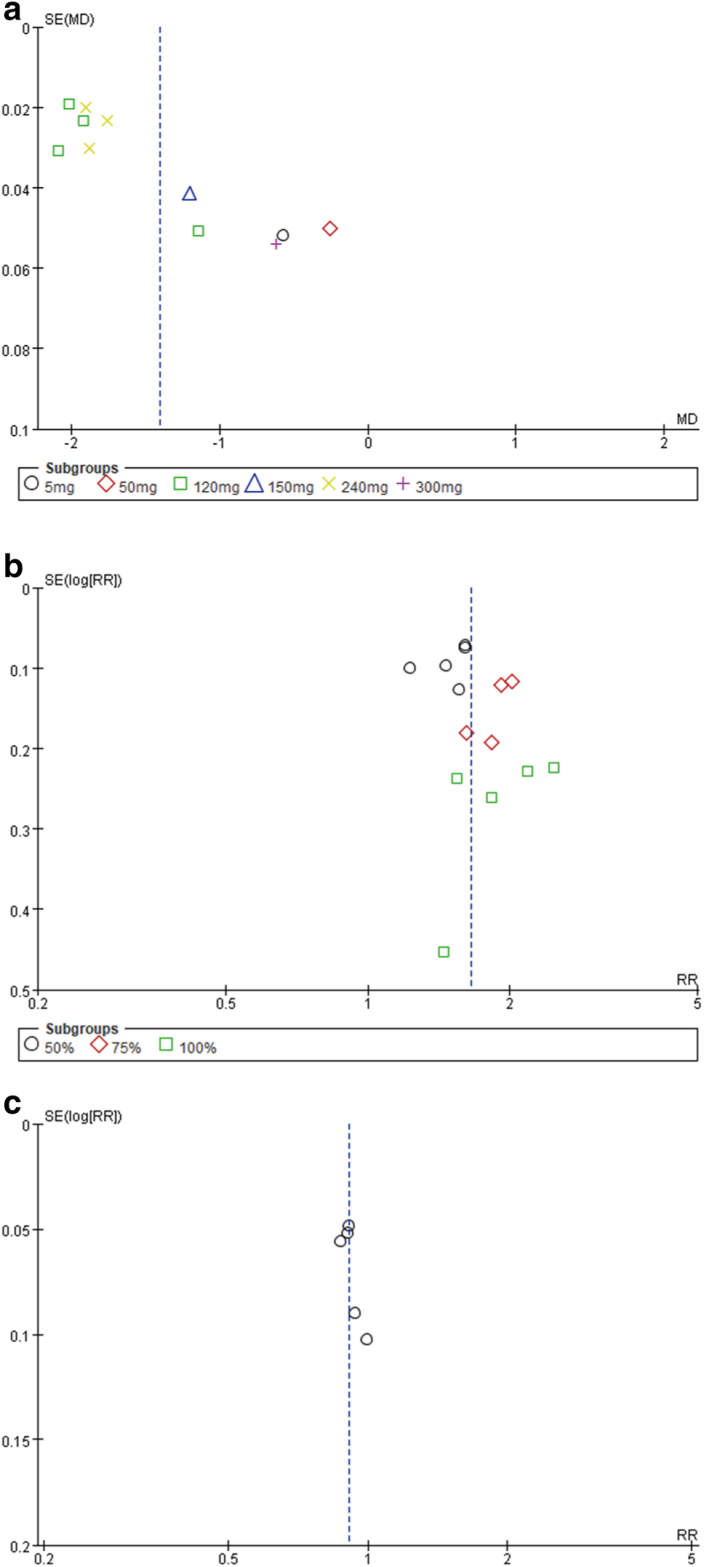
The development of anti-drug antibodies (ADA) to galcanezumab. *RR* risk ratio, *CI* confidence interval

The most frequently reported treatment-emergent adverse events (TEAEs) was injection-site pain. Otherwise, the results of the REGAIN, EVOLVE-1, and EVOLVE-2 trials revealed injection-site reactions, injection-site erythema, injection-site pruritus, and injection-site swelling at a greater rate in one or both treatment groups, when compared to placebo group. The other AEs were presented in Table 3. All studies reported serious adverse reactions (SAE), but none of these SAEs occurred in more than one patient. Therefore, no SAEs was induced by the study drug. Furthermore, there were no clinical meaningful changes in vital signs, ECGs, or laboratory parameters between galcanezumab and placebo. According to David W Dodick, it was only stated that 20 patients were detected with anti-drug antibodies (ADAs) at the end of the study, but the study did not clearly describe the antibody status in each group. Other ADA details are presented in Fig. 8 (Total: RR 2.89, 95% CI 1.74–4.80, *P* < 0.0001, *I*^2^ = 37%).

**Table 3:** Major adverse events (includes treatment-emergent adverse events)

Outcomes	Number of studies	Number of adverse events/participants		*I*^2^	Risk ratio (RR)	95% CI	*P* value
		Galcanezumab	Placebo				
Injection-site pain	5	205/1579	149/1419	60%	1.43	0.99–2.06	0.04
Nasopharyngitis	5	106/1579	104/1419	19%	0.91	0.67–1.24	0.56
Upper respiratory tract infection	4	93/1153	55/987	0%	1.33	0.97–1.83	0.08
Injection-site erythema	4	54/1306	20/1275	0%	2.44	1.46–4.06	0.0006
Back pain	4	47/1125	36/958	15%	1.03	0.64–1.66	1.03
Sinusitis	4	39/1125	26/958	0%	1.33	0.81–2.19	0.26
Influenza	4	34/1472	23/1309	0%	1.36	0.79–2.33	0.26
Neck pain	4	21/1125	14/958	0%	1.27	0.64–2.53	0.49
Dizziness	3	31/987	24/1003	0%	1.31	0.77–2.22	0.32
Nausea	3	24/806	29/679	0%	0.74	0.43–1.27	0.27
Injection site pruritus	3	39/1199	2/1172	0%	13.42	3.70–48.62	< 0.0001
Injection site reaction	3	67/1199	14/1172	73%	4.87	1.20–19.85	0.03
Urinary tract infection	3	35/1018	23/848	0%	1.39	0.83–2.35	0.21
Abdominal pain	2	16/426	12/389	0%	1.21	0.58–2.55	0.61
Arthralgia	2	12/426	12/389	0%	0.95	0.43–2.09	0.90
Dysmenorrhea	2	12/699	2/569	0%	3.32	0.80–13.71	0.10
Migraine	2	16/745	9/711	0%	1.66	0.74–3.73	0.22
Oropharyngeal pain	2	14/745	6/711	0%	2.20	0.85–5.68	0.10
Weight increased	2	13/745	10/711	0%	1.23	0.54–2.80	0.63
Fatigue	2	23/773	22/740	0%	0.99	0.55–1.76	0.97
Diarrhea	2	19/773	20/740	0%	0.90	0.48–1.60	0.74
Bronchitis	2	15/699	6/569	0%	1.92	0.74–4.95	0.18
Rash	1	5/107	0/110	–	11.31	0.63–201.99	0.10
Hypertension	1	5/107	0/110	–	11.31	0.63–201.99	0.10
Pain in extremity	1	4/107	5/110	–	0.82	0.23–2.98	0.77
Toothache	1	4/107	1/110	–	4.11	0.47–36.20	0.20
Viral gastroenteritis	1	2/107	4/110	–	0.51	0.10–2.75	0.44
Cough	1	10/426	7/432	–	1.45	0.56–3.77	0.45
Pruritus	1	8/426	1/432	–	8.11	1.02–64.58	0.05
Injection site bruising	1	6/426	6/432	–	1.01	0.33–3.12	0.98
Nasal congestion	1	6/426	4/432	–	1.52	0.43–5.35	0.51
Vertigo	1	6/426	2/432	–	3.04	0.62–14.99	0.17
Contusion	1	5/426	5/432	–	1.01	0.30–3.48	0.98
Injection site swelling	1	6/454	0/461	–	13.20	0.75–233.63	0.08
Pyrexia	1	6/319	2/279	–	2.62	0.53–12.9	0.24
Pain in extremity	1	3/273	1/137	–	1.51	0.16–14.34	0.72

## Discussion

### Effectiveness of galcanezumab

The meta-analysis evaluated the efficacy and safety of galcanezumab for the treatment of migraine. In this part of the analysis, 5258 patients were included. Monthly migraine days, headache hours, and the number of monthly migraine days that required acute treatment were all significantly lower than those from baseline. Furthermore, the ≥ 50%, ≥ 75%, or 100% response was greater in the galcanezumab group, when compared to placebo [12–17]. Further research should be performed for patients with 100% or no treatment response to identify predictors. Unilateral pain, unilateral autonomic symptoms, or allodynia have been considered as possible markers for those extremely good responders, but their predictive value still needs to be further confirmed [18].

The response in galcanezumab-treated patients could last for more consecutive months [19]. Efficacy even continued to exist during the post-treatment periods. Given the results from the randomized phase III trials, the therapeutic effect of galcanezumab was reduced after therapeutic treatment as a whole, but MMDs did not return to baseline [20, 21]. Galcanezumab not only lasted for a long time, but also had a rapid onset of action. According to the post hoc analysis of phase II-a study, the significant change in migraine headache days initiated an onset in the first week. Nearly half of the responses happened in the first month [22]. According to the EVOLVE studies’ subgroup analysis, the drug (galcanezumab) took effect a day after injection. It might be explained by pharmacokinetic characteristics of galcanezumab that an average time to its peak serum concentration was 5 days. Otherwise, double administration of the first dose helped to speed up the onset of the effects as well, since the therapeutic steady-state concentration of galcanezumab might be achieved after the first injection [23, 24]. Even if there was no notable initial effect, more patients would be relieved of headache through continuous administration [25].

Galcanezumab had effectiveness on individuals with failed preventions too. Differences in outcomes between galcanezumab and placebo were larger in the prior preventive failure subgroups based on the EVOLVE studies. It appeared to be driven by the lower placebo response in patients with prior failure [26]. The same conclusion was drawn from another post hoc analyses of 3 phase III studies, in which galcanezumab was provided as following treatment after failure to onabotulinumtoxinA. It's worth noting that our analyses did not compare the efficacy of galcanezumab to onabotA [27]. The data of head-to-head trials compared galcanezumab to oral preventatives are limited currently.

All results suggest that galcanezumab is effective for the prevention of migraine, but it is noteworthy that a high level of heterogeneity was found in the efficacy analysis. This may be because studies in the meta-analysis contained both episodic and chronic migraine population. Furthermore, the basic characteristics of each participant also more or less varied. However, these did not affect the conclusion.

The open-label phase study of REGAIN revealed that treatment with galcanezumab is likely to lead to high satisfaction with a therapeutic effect, together with meaningful reductions in health care resource utilization and acute headache medication [28]. This confirms that galcanezumab effectively improves the quality of life of migraine participants through direct or indirect contribution. The efficacy of galcanezumab was equal between those with high-frequency episodic migraine and those with low-frequency episodic migraine [29]. These above results are applicable to chronic migraine patients, whose previous migraine preventive treatments all failed [30].

### Safety of galcanezumab

The safety of galcanezumab was proven through minimal changes from baseline in vital signs, ECGs, and laboratory parameters. Among the phase II and III trials, no apparent differences in frequency and type of TEAEs were exposed between the galcanezumab dose groups and placebo group, except for EVOLVE-2, in which the galcanezumab 240 mg group exhibited a larger proportion of patients that referred at least one TEAE. Most of the TEAEs were transient, and mild or moderate in severity, without any obvious relationship with prolonged drug exposure, which was likely to be due to the long half-life of the Ab [31]. In addition to the studies included in the present meta-analysis, a phase III, long-term open-label study was performed to evaluate the safety and tolerability of galcanezumab. The findings supported those safety analyses in the other five previous studies [32].

Since CGRP can cause vasodilation, vascular adverse reactions deserve special attention. In the galcanezumab 240 mg group of EVOLVE-2, seven patients suffered from acute myocardial infarction and transient ischemic attack [15]. Meanwhile, hypertension was observed in five patients in the clinical trials, but it remains uncertain if these patients had hypertension before enrollment [33]. Although the results of these trials revealed that the administration of galcanezumab was not associated with a time- or dose-related cardiovascular events, it is necessary to verify these through long-term large-sample-size studies. On the other hand, it is a lack of evidence that galcanezumab was safe in those with known cardiovascular disease. Patients with acute or serious cardiovascular risks were excluded on account of the inclusion/exclusion criteria [34]. Galcanezumab exhibited low hepatotoxicity and nephrotoxicity in all test data which was probably because majority of the antibodies were eliminated via intracellular catabolism into the peptides and amino acids by endocytosis. However, the large volume of antibodies prevented these to be effectively filtered through the glomerulus [23].

Another concern for the safety of galcanezumab in these studies was treatment-emergent ADAs and neutralizing ADAs, which can increase or decrease the clearance of galcanezumab, or inhibit the ligand binding to it. Accordingly, the emergence of ADAs is correlated with possible allergic drug reactions, low efficacy, and AEs. Fortunately, studies revealed no impact on either safety or efficacy of galcanezumab by ADAs [31]. However, it should be noted that the immunogenicity results are highly dependent on the assay methodology, and this may be misleading on the comparison of the incidence of ADA across studies [23].

### Limitations of the meta-analysis

The present review also has some limitations. First, the present study was restricted to eligibility criteria, in which merely five studies were included in the analysis. Some unpublished and missing data of studies might also influence aggregate results. Furthermore, some of the studies were completed by the same researchers, which may lead to publication bias. In addition, the double-blind period of these present included studies ranged from 3 to 6 months, and the difference might result in heterogeneity. Finally, due to the exclusion of patients older than 65 years old, gravidas, or patients with a history of major cardiovascular or cerebrovascular diseases, the results of the systematic review lack universality.

Furthermore, studies with longer follow-ups and larger samples sizes should be performed to identify the confirmative safety profile of galcanezumab, and determine the duration of its therapeutic effects.

## Conclusion

The present meta-analysis systematically reveals that galcanezumab is superior to placebo for migraine, in terms of efficacy, safety, and tolerability. Indeed, these finding needs further investigations to identify the causes of the statistical heterogeneity among studies. However, overall, galcanezumab is a safe and well-tolerated pharmaceutical reagent that can be offered to migraine patients.
