# Correction to: Efficacy and safety of galcanezumab for preventive treatment of migraine: a systematic review and meta-analysis

**DOI:** 10.1007/s00415-020-10337-0

**Published:** 2021-02-08

**Authors:** Xiuyuan Zhao, Xiaolin Xu, Qingyun Li

**Affiliations:** 1 Tianjin Medical University, Tianjin, China; 2 Neurology, Tianjin Huanhu Hospital, Tianjin, China

## Correction to: Journal of Neurology 10.1007/s00415-020-09707-5

The authors would like to correct errors in the publication of their original article. The corrected details are given below:

On page 2 in the second paragraph, the date of first marketing authorization of galcanezumab needs to be corrected to September 2018. The correct sentence is:

“Thus, anti-CGRP monoclonal antibodies are used as the prophylactic treatments for migraine. Galcanezumab was authorized in the USA in *September* 2018.”

On page 10 in the fourth paragraph it is stated, that seven patients in the galcanezumab 240 mg group of EVOLVE-2 suffered from acute myocardial infarction *and transient ischemic attack*, while in fact the source cited states that one patient reported acute myocardial infarction and one patient reported transient ischemic attack. The correct sentence is:

“In the galcanezumab 240 mg group of EVOLVE-2, *one patient reported acute myocardial infarction and one patient reported transient ischemic attack* [15]”.

On page 10 in the fourth paragraph, the authors would like to delete the following phrase:

“Meanwhile, hypertension was observed in five patients in the clinical trials, but it remains uncertain if these patients had hypertension before enrollment [33].”

In the original text, the place that needs to see Fig. 5 is changed to Fig. 8. The place that needs to see Fig. 6 is changed to Fig. 5. The place that needs to see Fig. 7 is changed to Fig. 6. And the place that needs to see Fig. 8 is changed to Fig. 7.

Furthermore, in Fig. 5 the x-axis had to be moved to correctly reflect the data; please find the revised Fig. 5 here:

The revised Fig. 5 used the rigorous data published on the clinical trial website, and carefully checked the operation on Revman at each step.

As a result, the conclusion needs to be changed to “Our meta-analysis suggested galcanezumab had favorable influence on the increase in ≥ 50% response, ≥ 75% response, and 100% response. As for the control group also has a high ≥ 50% response, ≥ 75% response, and 100% response, the reason may be to comfort the psychological function of the patients."

Lastly, Figs. 6–8 have been mislabeled by accident; please find the corrected text references and figure labels below:

Figure 6 displays the adverse effects; the label for Fig. 6 should read:

“Fig. 6 Adverse events for galcanezumab (RR, risk ratio; CI, confidence interval)”.

Figure 7 displays the anti-drug antibodies data; the label for Fig. 7 should read:

“Fig. 7 The development of anti-drug antibodies (ADA) to galcanezumab (RR, risk ratio; CI, confidence interval)”.

Figure 8 displays funnel plots; the label for Fig. 8 should read:

“Fig. 8 Funnel plot of the publication bias”.

**Fig. 5:**
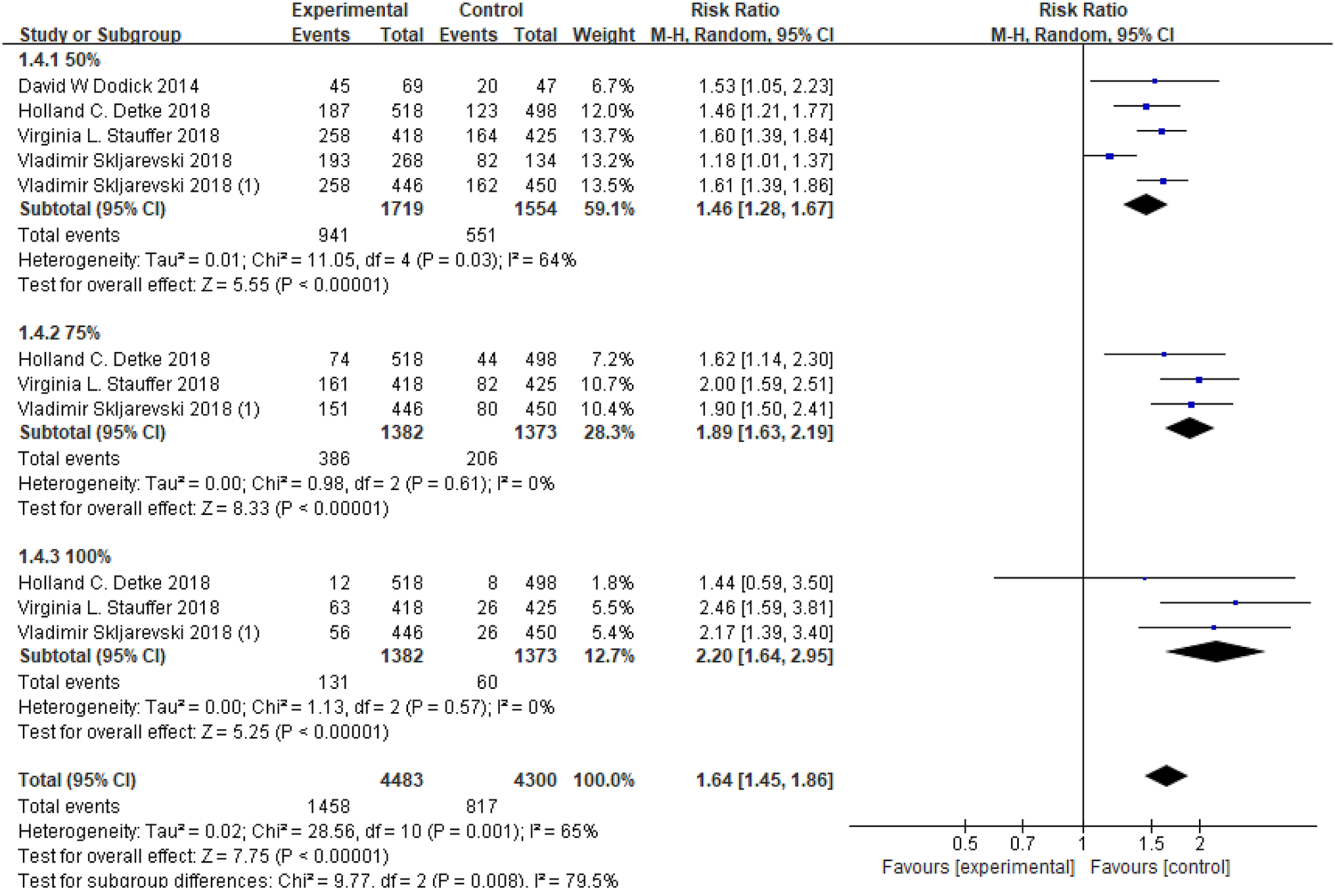
The 50%, 75% and 100% responder rates of the reduction from baseline in MMDs (RR, risk ratio; CI, confidence interval)

